# Torn between two lovers – on being a psychologist in a university medical centre

**DOI:** 10.1080/21642850.2023.2170379

**Published:** 2023-01-27

**Authors:** Ad A. Kaptein

**Affiliations:** Medical Psychology, Leiden University Medical Centre, Leiden, The Netherlands

**Keywords:** Medical psychology, university medical centre, outcome research, health humanities, history of health psychology

## Abstract

**Background:**

Psychology as applied to health and illness has a relatively short history. Nevertheless, that history shows a rapid development of the theoretical models that guide the field over the past 60 years. Core theoretical approaches are concisely reviewed, in the context of Kaplan’s paper ‘Behavior as the central outcome in health care’ (1990), which is used as a model to examine the extent to which these approaches embrace Kaplan’s notions.

**Advances:**

Empirical studies from the health psychology domain are used, which demonstrate the gains in terms of quality of life and behavioural outcomes in patients with (chronic) somatic diseases. Over a period of some 60 years, theoretical models and core concepts in psychology as applied to health and illness have evolved from psychosomatic views to neuropsychology, quality of life, patient education, self-management, illness perceptions, patient-reported outcome measures (PROMs), shared decision-making (SDM) and health humanities (HH). The more recent models (SDM, HH) appear to align to a considerable degree with adopting ‘behavior as the central outcome an outcome in health care’; shared decision-making and health humanities focus on encouraging patients to make sense of and give meaning to their illness in order to attain optimal psychosocial adjustment.

**Conclusions:**

In addition to ‘behavior as the central outcome in health care’, a new definition of the concept of health (i.e. ‘the ability to adapt and to self-manage’ – Huber et al., 2011) seems to favour patients, healthcare providers, society, and health psychology. Incorporating this concept into medical care may be viewed as a challenge for health psychologists – and as a source of continual struggle with strong biomedical forces.

It is dangerous to have two cultures which can’t or don’t communicate (Snow, 1964, p. 98)

She is 16 years of age. She has been rushed to the emergency room of a teaching hospital a few days ago because of acute severe asthma (*status asthmaticus*), in danger of dying. Stabilised a few days later, she has been transferred to the respiratory ward, where she is now in bed, connected to at least three IV-lines. During this morning’s grand round, the senior physician informed her of her situation. His three-minute monologue, spoken in a kind and supportive manner, bristles with words such as stimuli, dyspnea, pulmonary function, prevention, and inhalers. Two hours later I hear her approaching my tiny office, walking along in the hospital corridor, dragging her IV-pole along, breathing very audibly. She is meeting me to discuss her situation, and to fill out a set of questionnaires. The questionnaires are related to coping and living with asthma. When I explain the questionnaires to her, she responds angrily: ‘Asthma? I have no asthma whatsoever!’. When I ask her about what the physician told her two hours ago, she says she has not a clue. Some three weeks later she is discharged. Two months later an ambulance takes her to the emergency room again. The nurses greet her, she greets the nurses, they know each other quite well; the physicians sigh.

A week later, a 45-year-old male is admitted to the respiratory ward, in the middle of the night. Also with a very severe, life-threatening asthma episode. He is the author of several novels, quite well received in literary circles. When able to speak and walk, I meet him. He wants to study the manuals of the questionnaires, out of personal and intellectual curiosity. Listening to his story about what probably contributed to his hospital admission, an extended set of dramas unfolds: heavy alcohol use; divorce; writer’s block; complete derailment of his eating, drinking, and sleeping behaviour. The nurses avoid him due what they perceive as arrogance.

As a researcher, in a white coat, I note down my observations in the medical records of both patients. Those records are consulted many times a day by physicians and nurses. No one takes any notice of my notes. Laboratory data (e.g. blood parameters, pulmonary function) are studied in detail. Some two years later, a publication on the predictive value of illness perceptions for length of hospitalisation, risk of rehospitalisation, and severity of medication prescribed at discharge, summarises the findings from that study in the hospital ward (Kaptein, 1982)*.* At that time addressing illness behaviour and stimulating adequate self-management in order to reduce risk of hospitalisation are therapeutic approaches yet to be discovered – and studied by health psychologists.

The paper on illness behaviour in patients with asthma was my first publication in the medical psychology/health psychology domain – in a journal aptly titled *Social Science & Medicine*. Forty years later virtually every issue of major medical journals in the respiratory disease field contains papers on the self-management of asthma. The same goes for journals in virtually every other medical specialty (e.g. oncology, endocrinology, rheumatology, etc.). The history of health psychology in the UK is the focus of a paper by Quinn et al., 2020. The objective of this essay is to discuss some topics of the history of psychology as applied to health and illness – through the lens of someone whose research and teaching took place in a university medical school setting in a European country. It is a more or less personal account of a 40-year odyssey, studying the meaning of being ill and of how health psychology studies this subject.

When explaining to medical professionals what I focused on, I often used the term ‘behavioural medicine’; the term ‘health psychologist’ or ‘health psychology’ tends to lead to mild amusement and some bewilderment in a medical environment. The label ‘psychology as applied to health and illness’ works in social science and biomedical settings. This label was also used in one of the first journals in health psychology, *Psychology & Health* (volume 1: 1984, founding editor John Weinman). Looking back some 40 years, one can only express awe about the development of health psychology: societies have been established and prosper, and they encourage young researchers to help to further develop the field; an impressive number of journals specifically focusing on health psychology have been established, successfully, given their impact factors; books with an in-depth focus on specific health psychology topics have been published; conferences are fertile breeding grounds for exchanging research ideas and initiating international collaboration; research is translated into clinical care, instrumental in helping ill persons to enjoy the improved quality of life as the outcome of this care.

## Behaviour as the central outcome in health care (Kaplan, 1990)

The 1990 paper by Kaplan with the above title represents a core publication in health psychology and behavioural medicine. A number of sentences are too beautiful and too important to not quote them:

 … the only important outcomes in health and illness are behavioral. … Health outcomes are behavioral, and one way to improve them is to modify behavior. The behavioral conceptualization does not disregard the traditional medical model. Indeed, medicines and surgeries are excellent methods for improving behavioral health outcomes. However, the behavioral model is broader (p. 1218).

Given the importance that the medical world attaches to mortality, another quote is meaningful as well (and may be even somewhat humorous): ‘Death is a behavioral outcome. It can be defined as the point at which there is no observable behavior’ (p. 1212). One only has to examine papers in major medical journals to see how most of those papers are quite remote from ‘behaviour as the outcome in health care’: laboratory measures and readings from diagnostic instruments (e.g. MRI) make up the majority of outcomes in these papers. The absence of patient reported data is striking. This, by the way, seems true for much health psychology research as well. Quite a few papers in major health psychology journals appear to study outcomes that are quite a few miles away from ‘behaviour’ (Kaptein, 2011).

**Figure 1.**
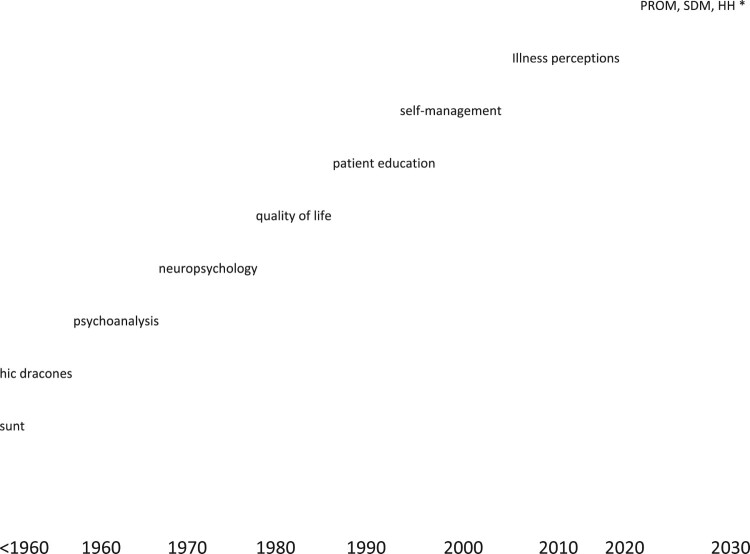
Theoretical approaches to health psychology research and clinical care for patients with somatic diseases, 1960 – present [*PROM = patient-reported outcome measures; SDM = shared decision-making; HH = health humanities].

It is fascinating to examine the degree to which behaviour is indeed the central outcome in healthcare in research performed by health psychologists over the past decades. Figure 1 attempts to sketch the changes over time. The figure depicts the various foci of research and clinical care in the health psychology field, based on a bird’s eye view of research at the time. The vertical axis represents the degree to which behaviour is the central outcome in healthcare, as evidenced in empirical papers in the health psychology domain. It would be rather arrogant to try and summarise the history of (health) psychology in one figure. Figure 1, therefore, merely attempts to sketch how major theoretical approaches in health psychology can be arranged chronologically, but also can be placed in a grid with a vertical axis that reflects the degree to which observable behaviour is conceptualised as an important outcome. The most recent theoretical models or approaches (patient-reported outcomes measures, shared – decision making, health humanities) may need more time and more empirical study before allotting them a more prominent place in the figure.

*Hic dracones sunt* (‘this is where the dragons reign’): in these dark times psychoanalysis dominated thought and practice regarding patients experiencing mental problems associated with physical problems. Psychosomatic medicine in the classical sense formulated ideas about how psychological factors were causally related to specific physical illnesses (e.g. asthma, hypertension). ‘Blaming the victim’ was rife.

Attempts to apply therapeutic methods that were part of psychosomatic theory to patients with – for instance – asthma were, therefore, of a psychiatric nature. In individual or group therapy, relations of patients with important others (mothers in particular) were discussed, aimed at addressing assumed problematic conflicts between the patient and relevant others. Effects of the interventions were assessed – if at all – in changes in self-reported psychodynamic constructs. Only when research psychologists studied the effects of behavioural therapeutic methods (e.g. Creer, 1979) were behavioural outcomes used as dependent variables, for example, activities of daily living, anxiety, and self-efficacy, in addition to absence from work or school.

Modern health psychologists use the concept of causal attributions in their research on illness behaviour as well, albeit in a different perspective, as the idiosyncratic explanations by a patient of his or her disease. Item 9 of the Brief Illness Perception Questionnaire illustrates the point: ‘Please list in rank-order the three most important factors that you believe caused your illness’. This line of thinking helped develop the link between causal attributions and interventions, aimed at trying to encourage patients to adopt constructive self-management skill, a topic which will be addressed later on when the Common Sense Model is discussed (Petrie & Weinman, 2012).

Decades after the heydays of psychosomatic theories, patients with various somatic diseases foster causal attributions that fit quite well with psychosomatic views. Sontag (1978) in her *Illness as Metaphor* stresses how psychological causal attributions (for cancer) often refer to ‘repressed emotion’. In his autobiographical novel, *Mars*, the Swiss author Fritz Zorn explicitly attributes his cancer to his psychosocial background:

I am young and rich and well-educated; and I am unhappy, neurotic and alone. I am a descendent of one of the best families from the right bank of Lake Zürich, also called the Gold Coast. I have been educated in an upper class environment and I have been cooperative all my life. My family is rather degenerated, and I am probably also rather genetically vulnerable and damaged. In addition, of course I also have cancer, as follows from this descent without saying (p. 25) … the cancer is just the bodily illustration of the situation of my soul (Zorn, 1979, p. 164).

### Neuropsychology

Still within the confines of the biomedical model, neuropsychology formed a bridge between somatic disease on the one hand, and behaviour of the patients afflicted by various diseases, on the other. Physicians could not help but notice the sometimes quite significant disconnect between *objective* severity of the disease and its behavioural and social consequences, which stimulated the search for causes of the disconnect. Cognitive deficits, disturbances in the processing of information and other neuropsychological concepts were studied in their potential contribution to the lack of concordance between objective and *subjective* severity of diseases. *Behaviour* in this approach refers to the impact of neuropsychological consequences of the disease on daily activities of patients.

Behaviour in persons with COPD, operationalised with self-report questionnaires on daily activities, was shown to be associated with scores on neuropsychological tests (executive functioning, memory tests, language tests, and visuospatial functioning) (Brunette et al., 2021; Fix et al., 1982). In patients with asthma, a meta-analytic review of cognitive impairments revealed negative effects of the illness on academic achievement and executive functioning in particular (Irani et al., 2017).

Neuropsychological studies paved the way for studying how patient-related factors in the psychological sense impacted on the behavioural consequences of somatic disorders, helping introduce the concept of quality of life.

### Quality of life

Quality of life (QOL), defined in various ways, was – and probably still is – the magic word in the world of clinical medicine that opens doors for (health) psychologists in the medical arena. Physicians sincerely interested in ‘the effects of an illness and its treatment, as perceived by the patient’, began involving health psychologists to study QOL. Physicians baffled by the rather poor association between objective severity of a disease and the subjective response by patients tend to use QOL as a way to maintain the validity of objective severity of an illness (or rather, a disease).

QOL probably represents the most important area of (clinical) health psychology in research and clinical care in a medical setting. QOL is the core area of an impressive number of scientific journals, societies, and organisations (e.g. EORTC). Medical professionals are easily convinced of the importance and relevance of QOL in clinical care and research. A promising development in this field of research and clinical care pertains to incorporating QOL scores as complementary primary endpoints in medical care, allowing clinicians to counsel patients about QOL outcomes and clinical outcomes simultaneously (Gebski et al., 2022). However, a caveat seems in order here. QOL may function as a ‘fig leaf’ for the ills of a biomedical approach, if reduced to a number on a scale or questionnaire*.* In discussions about designing a study in a medical setting where all agree QOL should be assessed, (bio)medical researchers do not blink an eye when agreeing on a wide range of biomedical measures (e.g. blood characteristics, blood pressure, imaging techniques, physical examinations, etc.). If it is agreed that QOL should also be assessed, the pressure of biomedical colleagues to use a (very) short measure to assess QOL can be tough to resist. A commonly used measure to assess QOL (i.e. SF-36) – that may be better qualified as a measure of functional status – may even be reduced to the SF-6: the very reduced version of six items of the SF-36. QOL is then seen as a concept similar to Hb (blood) or mmHg (as a measure of blood pressure). QOL reduced to a fig leaf: the patient’s narrative reduced to a single digit. One cannot help but shiver when hearing a medical professional maintaining that ‘SF-6’ is a measure to assess QOL (e.g. Yong et al., 2020).

The journal *Quality of Life Research* and the ISOQOL (International Society for Quality of Life Research) are two major outlets for research, clinical care, and international collaboration in the area of quality of life research. The area of QOL might benefit from addressing some issues that may be somewhat problematic. Debates on defining the concept of QOL are still going on, as are discussions about the position and relevance of biomedical characteristics of the disease and the afflicted patients in the QOL-concept. Combining disease-generic and illness-specific QOL-measures seems to be a useful strategy. Furthermore, incorporating suggestions about how to use QOL-scores in interventions – also by medical and nursing staff – must be viewed as helping patients improve their QOL.

### Patient education

Research in the context of quality of life makes it clear that the impact of a disease and its medical treatment can be assessed with valid and reliable tests that come from other domains than the biomedical domain. This finding – not really surprising to behavioural scientists – initiated further research into determinants of QOL and work that aimed at changing – better said, improving – QOL. One approach consisted of ‘patient education’ to improve patient-physician communication and interaction, in the assumption that improving this communication would lead to improvements in QOL. Also, patient education was – and is – viewed as a method to provide patients with knowledge and information about a disease and its medical treatment, with the assumption that more information and knowledge helps patient achieve a better outcome. The journal *Patient Education and Counseling* is one of the major sources for empirical research in this area.

Sad examples of a biomedical view on providing patients with information in order to improve their disease status are easy to find. Robinson et al. (2018), for example, reported that adherence to adjuvant endocrine therapy in women with breast cancer, a very important intervention method to reduce morbidity and mortality after a breast cancer diagnosis, diminished quite dramatically after a few years, implying the need to develop interventions that help increase this adherence. Systematic reviews and meta-analyses abundantly show that ‘providing information on the necessity to be adherent’ are almost completely missing their target in this population (Kaptein et al., 2021). Nevertheless, the mantra in almost all studies in this area is, ‘this study offers support for the need to develop interventions such as reminder letters or telephone calls to enhance persistence rates, especially when patients reach the later years of adjuvant endocrine therapy’ (Robinson et al., p. e14).

**Figure 2.**
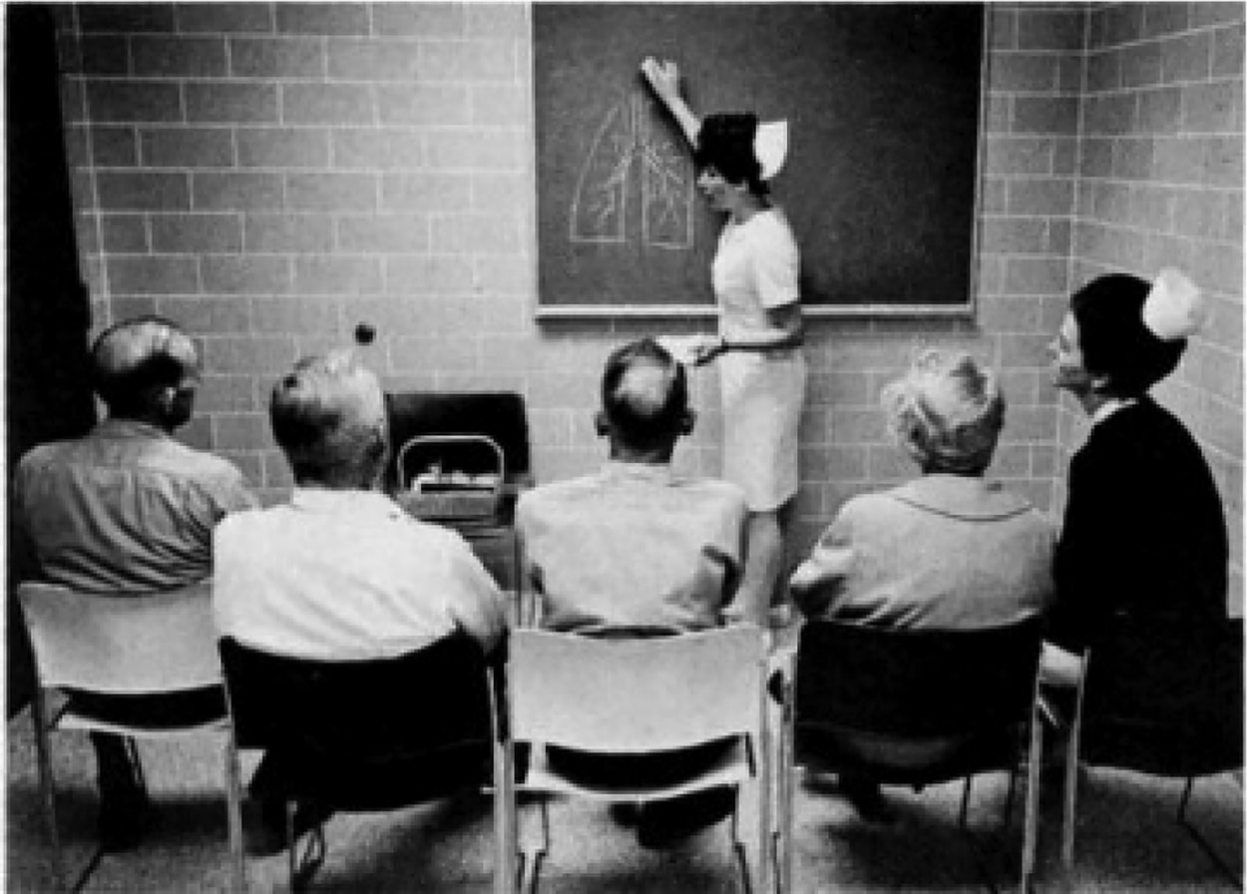
Patient education, “19th century style” (Neff & Petty, 1971).

Patient education has the connotation of the patient as a passive recipient of biomedical information, focusing on biomedical issues of a disease. Figure 2 says it all: the picture shows passive patients (with COPD), listening to a medical professional in a white coat, drawing on a black board biomedical issues about breathing. The picture is dated; nevertheless, papers in biomedical journals in 2022 make clear how the biomedical thinking to patient education still adopts this approach in order to ‘educate patients’.

**Figure 3.**
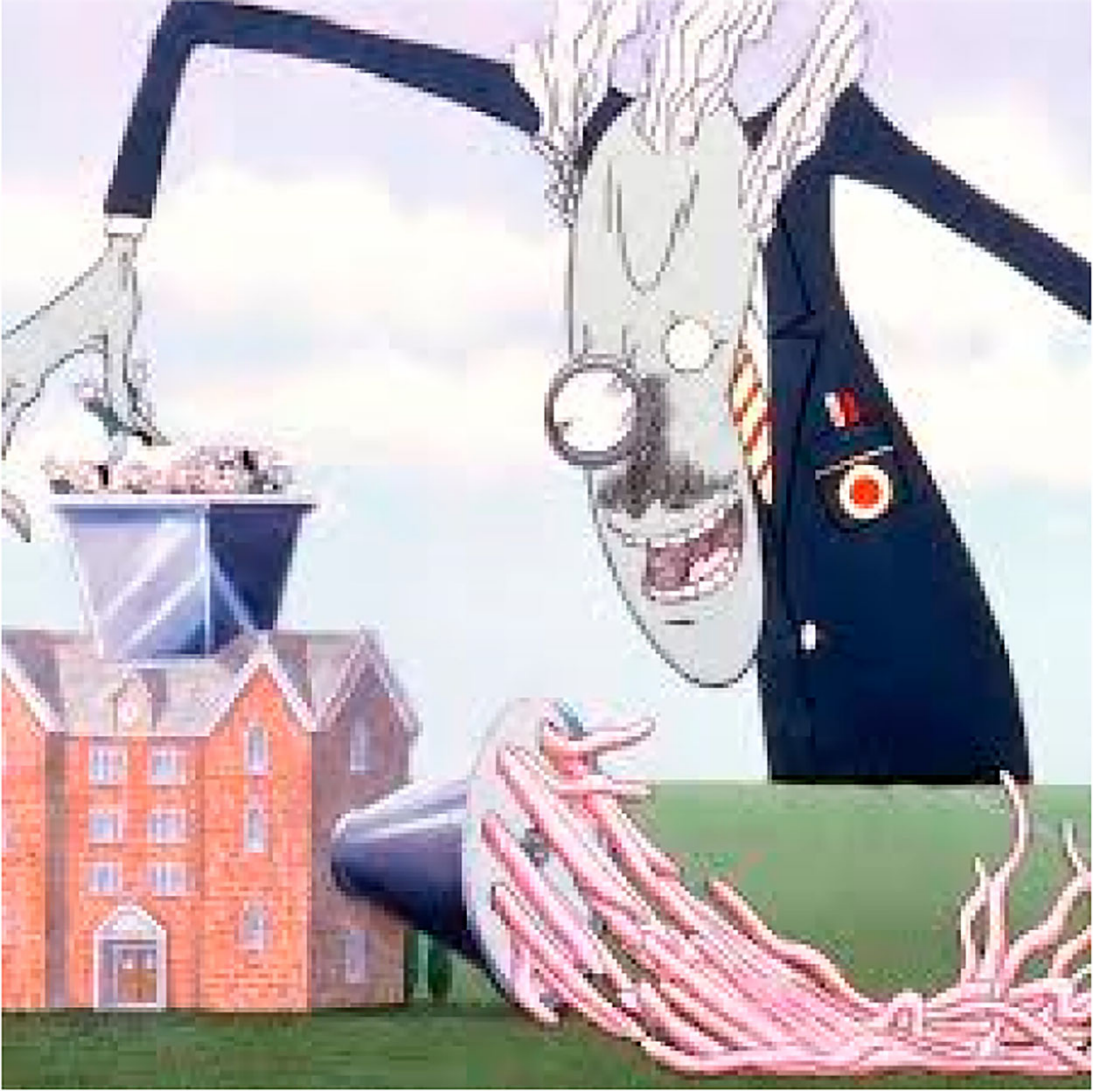
‘We don’t need no education’ – patient education old style (Kaptein et al., 2021).

With these observations, a turning point in efforts (by health psychologists in particular) seems to have been reached in helping patients with (chronic) somatic disease to live a better life, with as minimal an impact of the consequences of the illness in daily life as possible. Patient education in essence represents biomedical thinking: the patient must adopt the biomedical views about an illness (e.g. causes, treatment, prognosis, etc.) after which things will get better. Views of patients themselves about causes, treatment, prognosis etc. are considered ‘subjective’, ‘unscientific’ views of ‘lays’*.* Patient education in its early phase adopted a biomedical approach: patients were encouraged in attempts to make them ‘apprentice-physicians’. Examining papers in more recent issues of for instance the journal *Patient Education and Counseling* makes clear how such a biomedical emphasis has evolved into a biopsychosocial approach. In recent studies where patients are helped to live with their illness, patients’ cognitions and emotions are addressed and modified to be more in accordance with biomedical views, in order to promote better self-management (cf. the Petrie et al., 2002, study on addressing cognitions and emotions in patients after a myocardial infarction. See also the study by Jansen et al. (2013) where illness perceptions and treatment beliefs of people on hemodialysis were challenged and changed and brought in alignment with current nephrological expertise)*.* In an attempt to mock this view, the subtitle, ‘We don’t need no education’ was added to a paper on adherence to targeted therapy (Figure 3; Kaptein et al., 2021). The Editor of the journal deserves praise for her decision to leave that subtitle untouched.

Recently the *Journal of Patient Education* is starting to pay attention to more advanced approaches to involving patients in biopsychosocial care: see the papers on patient – reported outcome measures (PROMs) and shared decision-making (SDM) in the most recent issues. Developments towards combining providing information with encouraging some sort of ‘self-management’ (the issue discussed in the next paragraph) are becoming visible. Hwang et al. for instance report a randomised controlled trial in patients with heart failure (Hwang et al., 2022). Patients in the experimental condition received an individual nurse-led education session on heart failure self-management and three telephone calls after discharge. Improvements in not only knowledge and quality of life but also in self-care were found.

### Self-management

Self-management of chronic somatic illness is defined as

 … the individual’s ability to manage the symptoms, treatment, physical and psychological consequences and life style changes inherent in living with a chronic condition … to monitor one’s condition and to effect the cognitive, behavioural and emotional responses necessary to maintain a satisfactory quality of life (Barlow et al., 2002, p. 178).

Barlow et al. list seven components of self-management: contrary to what most medical professionals assume, gathering information is only one of those seven components. The other six refer to behavioural skills in particular: managing medication, managing symptoms, managing psychological consequences, adjusting lifestyle, using social support, and communicating effectively. Compared to patient education, self-management therefore represents one step further in the direction of the patient as an active participant in the management of a somatic condition.

Self-management would have been quite helpful to the two patients described in the beginning of this paper. A systematic review of the effects of strategies to support self-management in asthma concludes that ‘behavioral support on asthma self-management more often than once a month with an aid of e-Health could improve asthma control, whereas interaction with health care provider helps to lessen the risk of asthma exacerbation’ (Dhippayom et al., 2022, p. 803). The systematic review by Hodkinson et al. (2020) concisely concludes that ‘ … regularly supported self-management reduces the use of healthcare resources and improves quality of life across all levels of asthma severity’ (p. 1). In the respiratory diseases category, recent reviews on self-management for patients with COPD (chronic obstructive respiratory disease) report similar relatively positive findings. For instance: ‘ … the results support psychosocial intervention as an additional, useful tool in multidisciplinary respiratory care … to improve both psychological and physical outcomes’ (Farver-Vestergaard et al., 2022, p. 347). Healthcare providers used to adopting biomedical views may be more convinced of the value of self-management in patients with COPD by the findings of a recent Cochrane review – reviews that are usually quite biomedically biased – that concludes that ‘ … self-management interventions for people with COPD are associated with improvements in health-related quality of life … and a lower probability of respiratory-related hospital admissions’ (Schrijver et al., 2022, p. 2).

In all studies used here as illustrations of self-management, behavioural outcomes are prominent, rather than ‘distal’ outcomes such as pulmonary function or 6-minutes walk test (let alone ‘intentions’ to exercise daily; Kaptein, 2011).

### Common Sense Model – illness perceptions

Patient education research helped stimulate the study of patients’ views of their illness, encouraging patients to incorporate self-management skills into living with an illness. The meaning of the illness – in the cognitive and emotional sense – became a major object of study in clinical health psychology. The Common Sense Model (CSM) is the hallmark of this approach (Leventhal, 2019). An impressive number of excellent reviews are available on the CSM, theoretically and empirically (e.g. Hagger & Orbell, 2022). The study by Petrie et al., 2002, which has a strong experimental design, is still exemplary in demonstrating that the effects of addressing illness perceptions and changing them into more adaptive ones in patients with a myocardial infarction led to changes in behavioural outcomes: return to work, resumption of sexual activities, and (self-reported) improvements in coping. More recent publications corroborate these findings (Alyami et al., 2021; Breland et al., 2020; Chan et al., 2021; Hayes et al., 2020).

In an ambitious systematic review, Breland et al. (2020) examined the associations between the CSM constructs and the construct of self-efficacy, with self-management behaviour and health outcome in studies of adults with chronic somatic conditions. Both categories of constructs were found to be associated with the two dependent variables, with the CSM being particularly useful when trying to understand health outcomes (i.e. symptoms, QOL, illness specific status), and self-efficacy in its associations with self-management behaviour (i.e. exercise, diet, medication adherence).

‘Death is the absence of observable behavior’ (Kaplan, 1990, p. 1212): given this dictum, van Dijk et al. (2009) studied the relation with mortality of patients’ illness perceptions of their end-stage renal disease. Scores on the Illness Perception Questionnaire – Revised, especially perceptions of treatment control, prospectively predicted mortality.

The girl of 16 and the man of 45 with status asthmaticus described in the Introduction of this paper exhibited illness perceptions – a concept that did not exist as such at the time. The CSM proves to be a fertile ground for research in a medical university setting. Our research group has been able to help explore the area of illness perceptions in adults with chronic somatic disorders (e.g. endocrinology [Andela et al., 2015]; rheumatology [Bijsterbosch et al., 2009]; respiratory diseases [Fischer et al., 2010]; neurology [Helder et al., 2002]; nephrology [Jansen et al., 2013]; oncology [van der Kloot et al., 2016]; ENT [Vogel et al., 2008]).

Clinical applications of the CSM are easily available, with overall quite encouraging results (e.g. the PubMed search www.pubmed.gov ‘Illness perceptions intervention AND systematic review’).

The CSM enjoys a great degree of empirical study (e.g. see Hagger & Orbell’s, 2022 conceptual review). Suggestions for further development of the model are given by these authors. They pertain, in particular, to issues that also seem relevant in QOL-research: ‘ … research that focusses on incipient illness samples, utilizes designs that capture dynamic process in the model, … and illness specific measures of coping’ (p. 347).

### Patient-reported outcomes measurement

Patient-reported outcomes are defined as ‘ … any report of the status of a patient’s health condition that comes directly from the patient, without interpretation of the patient’s response by a clinician or anyone else’ (Meadows, 2022, p. 1703). The concept of patient-reported outcome measurement (PROM) does not seem miles away from the concept of QOL – judging from recent papers on the effects of assessing PROMs in clinical care. PROMs, however, do still seem to have a somewhat biomedical emphasis. Meadows correctly notes ‘how the patient’s illness narrative is lost along the way’ (p. 1703). Nevertheless, recent publications and editorials in major medical journals (e.g. JAMA) show how the importance of ‘the status of patient’s health condition’ is increasingly recognised in routine medical care (Calvert et al., 2019; Rivera et al., 2019; Weinfurt & Reeve, 2022). This is also reflected in *The Journal of Patient-Reported Outcomes*, which publishes a respectable number of solid studies on PROMs.

### Shared decision-making

The approaches to clinical care and the associated position of the patient, outlined in Figure 1, share one characteristic: they all have the patient as the object of intervention. Although a shift is visible in the power balance between patient and healthcare provider over the past decades, interventions – also in the area of health psychology and behavioural medicine – run the risk of ‘blaming the victim’. It is the patient who is supposed to change his/her (illness) behaviour to improve the patient’s QOL. It would behoove healthcare providers (MDs, nurses, physiotherapists, and clinical health psychologists (sic)) to think about the power balance between patient and healthcare provider.

As such, shared decision-making (SDM), as the concept suggests, is an attempt to incorporate the patient’s views into decisions about the medical management of a complaint, symptom, or disease. It is ‘a patient engagement model that includes strategies that promote patient and clinician engagement to jointly consider management options … the clinician’s role is to facilitate discussion of the risks and merits associated with options, the goal of reconciling differences.’ (George et al., 2021, p. 1502).

Clinical health psychologists tend to view SDM positively as the approach implies incorporating ‘the patient’s views and the patient’s story’ into the medical management of a medical condition. In a Special Issue about SDM, the editor of the journal *Patient Education and Counseling* points out that ‘SDM is among the fastest growing areas of study in clinical communication research’ (Finset & Street, 2022, p. 1055). Also mentioned are a number of challenges associated with implementing, assessing, and measuring outcomes in the SDM approach. Healthcare providers’ reluctance to fully implement SDM is one problem; assessing the effects of SDM in medical encounters another. Similar findings are reported in a systematic review of SDM (Mathijssen et al., 2020). Nevertheless, SDM does imply that patients themselves are involved in defining what is important to them, in medical care, including the effects of medical care on patient’s behaviour.

### Health humanities

Health humanities (HH) is defined as

… a field concerned with understanding the human condition of health and illness in order to create knowledgeable and sensitive health care providers, patients, and family caregivers. As a field the health humanities draws on the methodologies of the humanities, fine arts and social sciences to provide insight, understanding, and meaning to people facing illness including professional care providers, lay care providers, patients, policy-makers and others concerned with the suffering of humans (Klugman & Lamb, 2019, p. 3).

Narrative Medicine can be conceptualised as a precursor of Health Humanities. Defined as ‘a framework for medicine and health sciences that values the individual’s stories and experiences as integral aspects of the lived experience of health and illness’ (Remein et al., 2020), Narrative Medicine encourages medical professionals to write about what patients tell them, and incorporate those stories in the medical management of patients. Also, Narrative Medicine encourages health professionals to engage with literature and art.

Garden (2015) puts health humanities in a psychological – and political – perspective: ‘they focus on suffering, rather than pathology, and on sociocultural understandings of illness and disability, rather than a narrow biomedical perspective. The health humanities thus analyse and attempt to recalibrate the power imbalance in health care’ (p. 77).

Similar to the shift from ‘medical psychology’ to ‘health psychology’, ‘medical humanities’ as a label is currently being replaced by the label ‘health humanities’. This is more than just word play as explained by Jones et al. (2017) in their paper ‘The almost right word’: health humanities encompass a broader set of concepts than concepts from the medical world.

**Figure 4.**
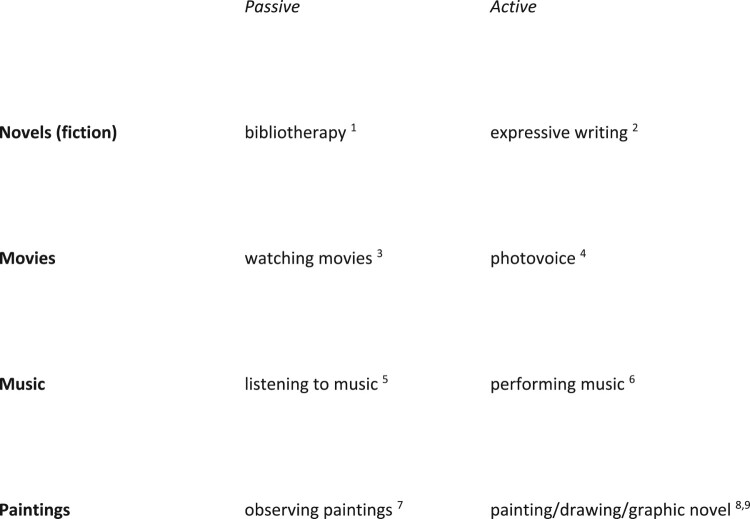
Health humanities: applications of four genres, with patient in passive or active role. [1 Bravender et al., 2010; 2 Pennebaker, 2018; 3 Jones et al., 2021; 4 Rayment et al., 2019; 5 van der Heijden et al., 2019, 6 https://disabilitymovies.com/2011/benda-bilili-2; 7 Quan et al., 2016; 8 and 9 Broadbent et al., 2019; Williams, 2012].

In a paper that aims at putting health humanities into a health psychology perspective, I outlined four art forms and their application to healthcare, distinguishing between an *active* and *passive* position of the patient, where passive does not necessarily mean that the patient is merely receiving an intervention (Figure 4; Kaptein, 2021, 2022; Kaptein, Hughes, Murray, & Smyth, 2018).

Health humanities offers art forms that allow ill patients to express the consequences of their affliction, and to vicariously observe how other patients express their illness and its medical management. For instance, reading a novel about asthma may offer the reader access to the world of living with asthma and coping with the respiratory disease. This passive form of using fiction can be contrasted with active form, ‘expressive writing’. In a major study, Smyth et al. published the results of an expressive writing experiment in patients with asthma (Smyth et al., 1999; for an update, see Valtonen, 2021). The authors found a statistically and clinically significant effect of expressive writing on pulmonary function. In the context of the current paper, there is a wealth of research and clinical material to help ‘give patients voice lessons’ (Frank, 1995), with empirical work supporting the use of health humanities in care for persons with chronic somatic illnesses. Theory of Mind (ToM; Kidd & Castano, 2013) and PNI (psychoneuroimmunology; Antoni & Dhabhar, 2019; Chang et al., 2022) are suggested as two theoretical contexts for HH. A study by Fancourt and Steptoe (2019) offers additional theoretical and empirical embedding of HH (see also Stephens, 2011).

Reading, expressive writing, watching movies, making movies, listening to music, performing music, observing paintings, painting – all activities encourage patients to give meaning to symptoms, illness, and medical management – thereby encouraging self-management, via constructing illness perceptions and treatment beliefs, as outlined in the CSM.

Responses by health professionals to patients who are actively or passively linking their illness to various art genres differ. Some smile somewhat condescendingly and mumble words such as ‘subjective’, ‘unscientific’. Others actively encourage patients to use various art forms to help patients give their illness a place in their lives. Given the strong dominance of the biomedical model in current healthcare, health humanities represent merely a niche. One of the major medical journals (JAMA) publishes a ‘Patient page’ in every issue; however, the page merely presents biomedical information to patients, demonstrating that biopsychosocial care is not yet mainstream in major medical outlets. The same goes for major medical textbooks used in medical schools. Health psychologists possess the research and clinical expertise to help things change to the better, one would hope.

## What I wish I had known … some closing remarks

It would be fascinating to see how another author, 40 years from now, would analyse the development of psychology as applied to health and illness. I have been fortunate enough to have witnessed the area develop over the past 40 years. Strong attempts to improve the position of the patient in the medical world are one of the consistent trends in those 40 years.

My quest for studying the lived reality of being ill has led to my stay of some four decades in health psychology (although that concept usually is greeted with shock and laughter in medical circles) – psychologists active in a medical setting prefer Medical Psychology. When talking with health psychologists based in a social science environment, I am somewhat surprised by their reluctance to consider (bio)medical issues in their research and clinical activities. When talking with physicians and (bio)medical students in a medical school setting, I am somewhat surprised by their resistance to and disgust of health psychology concepts, methods, and research results. ‘Never the twain will meet’ is an easy and rather sad summary of the situation in 2022 – and I think it must get worse before it gets better.

Dissatisfied by research using self-reported data on health behaviour and illness behaviour, I turned to two novel domains and methods of data-collection: drawings and novels (i.e. fiction). In their clinical work, MDs quite often use drawings of a medical condition of a patient or of a diagnostic or therapeutic procedure that the MD is proposing. These drawings tend to be highly technical, biomedical, and devoid of psychological, sensory information; they do not include any attention to the patient’s illness perceptions. Pharmaceutical companies even hand out pre-printed schematic diagrams of, say, heart or lungs, to help explain the medical situation in the patient-doctor encounter. Although the history of using drawings by patients in (health) psychology research is quite long, Broadbent must be credited with exploring the topic of drawing in health psychology (Broadbent et al., 2019). The 2019 review shows how drawing research now covers almost any medical condition, giving room to patients to express their illness and its consequences in daily life.

‘Novels as data – health humanities and health psychology’ (Kaptein, 2022) is an attempt to translate the writings of authors of novels about being ill into health psychology concepts, theory, and methods. I find Alexander Solzhenitsyn’s *Cancer Ward* much more informative (and beautiful and interesting) than any paper in a journal on biopsychosocial oncology. The corpus of novels, films, music, and paintings in relation to health and illness offers an exciting wealth of data that, when explored with health psychology theory and methodology, would contribute enormously to the understanding of the world of illness, with associated benefits for those ‘who are in the kingdom of the ill’ (Sontag, 1978).

‘What if I had known’ – I have been extremely fortunate and lucky in meeting a group of colleagues whose friendship, intellectual inspiration and research collaboration have helped shape my almost 50-year journey in the fascinating world of medical psychology and health psychology. The European Health Psychology Society (EHPS), with its associated journal *Psychology & Health*, have shaped my professional life.

Looking back some 50 years allows me to witness how health psychology has developed quite spectacularly. It is a privilege to have read thousands of manuscripts and papers in the health psychology field, to have read thousands of abstracts at conferences from young researchers on abstract boards. ‘Meet the professor’ sessions at conferences sometimes turned out to be sessions where the young participants’ main question would be ‘how do I become a professor ASAP?’ Successful researchers in academic health psychology, in my experience, are expert listeners to patients and their caregivers, and to health professionals, where they pick up innovative research and clinical ideas from their stories. They are able to collaborate closely with patients and their caregivers, and their physicians and nurses. They know how to steer away from superficial statistical models, know how to separate the wheat from the chaff (i.e. medicine from clinical epidemiology and evidence-based medicine). Incorporating the patient story into research and clinical work is the bedrock of the work of an academic health psychologist.

Arthur Frank (sociologist, 1995), Arthur Kleinman (psychiatrist and cultural anthropologist, 1988), and Howard Leventhal (psychologist, 2019) inspired and continue to inspire my thinking about making sense of illness. Making sense of illness is a fairly recent phenomenon, as explained by Armstrong (1984). His analysis of the birth of ‘the medical gaze’ explains some of the background of one of ‘the lovers’, i.e. the biomedical model:

Under the old medicine, signs and symptoms are and say the same thing … every symptom was a potential sign and the sign was simply a read symptom … in the new perception sign and symptom were separated: the symptom might well remain silent, the truth of the disease was contained only in what the doctor found, in the form of the sign. Symptoms, what the patient said, could provide a hint or a suspicion of which organ or system might be involved but were only preliminaries; the core task of medicine became not the elucidation of what the patient said but what the doctor saw in the depths of the body (Armstrong, 1984, p. 738).

**Figure 5.**
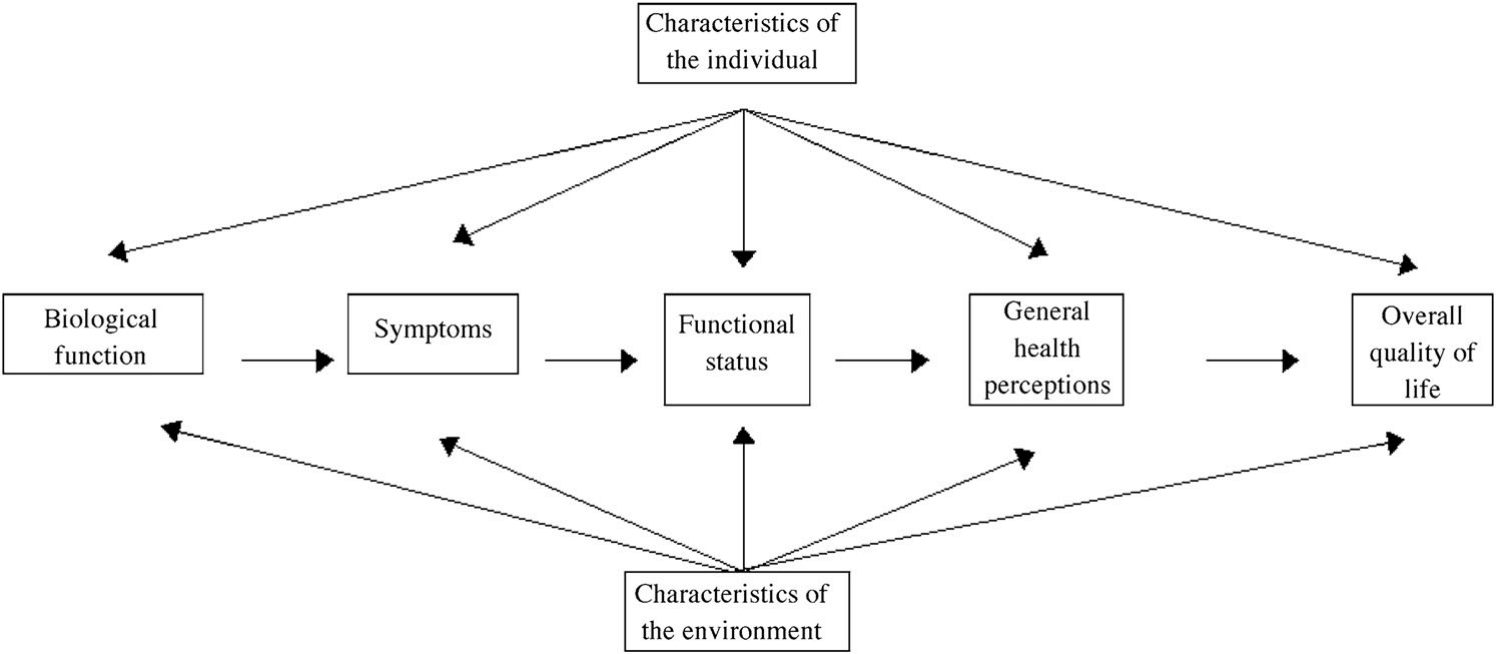
Conceptual model of health-related quality of life (Ferrans et al., 2005, p. 338).

In their paper ‘Linking clinical variables with health-related quality of life,’ Wilson and Cleary (1995) attempt to bring the ‘two lovers’ together in one model; Ferrans et al. (2005) further simplified that model (Figure 5). One would hope that the model is a home for both lovers: the biomedical and the psychological model.

Given Kaplan’s ‘Behavior as the central outcome in health care’, it is extremely encouraging to study the paper by Huber et al. (2011) entitled ‘Health – how should we define it? – with the answer “*Health is the ability to adapt and to self manage*”’ (p. 237). Health psychology does seem to have a bright future ahead of it.

The 16-year-old girl with asthma was referred to an asthma centre, where she participated in a self-management course. She was able to adopt illness perceptions that impacted positively on her quality of life. Supported by a move to a new, dry, and modern house, she managed to stay out of hospital care for a substantial period of time (Achstetter et al., 2019).

The 50-year-old male patient with asthma was discharged after three weeks. His autobiography, *The Fall* (1991), is a dramatic account of his demise. He passed away in 2007, at age 71 years.

## Data Availability

There are no data associated with this article. The study received an exemption from an Institutional Review Board/Ethics committee.
